# Optimized Machine Learning Model for Predicting Compressive Strength of Alkali-Activated Concrete Through Multi-Faceted Comparative Analysis

**DOI:** 10.3390/ma17205086

**Published:** 2024-10-18

**Authors:** Guo-Hua Fang, Zhong-Ming Lin, Cheng-Zhi Xie, Qing-Zhong Han, Ming-Yang Hong, Xin-Yu Zhao

**Affiliations:** 1 CCC-FHDI Engineering Corp., Ltd., Guangzhou 510290, China; fangguohua9999@163.com (G.-H.F.); linzhongming8202@163.com (Z.-M.L.); 2 China Construction Fourth Engineering Division Corp., Ltd., Guangzhou 510075, China; pcxcz88@163.com (C.-Z.X.); hccbz819@163.com (Q.-Z.H.); 3 State Key Laboratory of Subtropical Building and Urban Science, South China University of Technology, Guangzhou 510641, China; xbmhongmingyang@163.com

**Keywords:** alkali-activated concrete, data processing, data analysis, explainable machine learning models, optimization

## Abstract

Alkali-activated concrete (AAC), produced from industrial by-products like fly ash and slag, offers a promising alternative to traditional Portland cement concrete by significantly reducing carbon emissions. Yet, the inherent variability in AAC formulations presents a challenge for accurately predicting its compressive strength using conventional approaches. To address this, we leverage machine learning (ML) techniques, which enable more precise strength predictions based on a combination of material properties and cement mix design parameters. In this study, we curated an extensive dataset comprising 1756 unique AAC mixtures to support robust ML-based modeling. Four distinct input variable schemes were devised to identify the optimal predictor set, and a comparative analysis was performed to evaluate their effectiveness. After this, we investigated the performance of several popular ML algorithms, including random forest (RF), adaptive boosting (AdaBoost), gradient boosting regression trees (GBRTs), and extreme gradient boosting (XGBoost). Among these, the XGBoost model consistently outperformed its counterparts. To further enhance the predictive accuracy of the XGBoost model, we applied four state-of-the-art optimization techniques: the Gray Wolf Optimizer (GWO), Whale Optimization Algorithm (WOA), beetle antennae search (BAS), and Bayesian optimization (BO). The optimized XGBoost model delivered superior performance, achieving a remarkable coefficient of determination (R^2^) of 0.99 on the training set and 0.94 across the entire dataset. Finally, we employed SHapely Additive exPlanations (SHAP) to imbue the optimized model with interpretability, enabling deeper insights into the complex relationships governing AAC formulations. Through the lens of ML, we highlight the benefits of the multi-faceted synergistic approach for AAC strength prediction, which combines careful input parameter selection, optimal hyperparameter tuning, and enhanced model interpretability. This integrated strategy improves both the robustness and scalability of the model, offering a clear and reliable prediction of AAC performance.

## 1. Introduction

Concrete, an indispensable material in the construction industry, has become one of the most widely used materials worldwide. Among its variants, ordinary Portland cement concrete (OPCC) dominates, with ordinary Portland cement (OPC) production exceeding 4.4 billion tons annually [1,2,3,4]. However, the environmental toll of OPC is becoming increasingly concerning, contributing to approximately 5–8% of global anthropogenic CO_2_ emissions [5]. This has driven growing interest in alternatives to OPCC [6,7]. One of the most viable solutions is the use of alkali-activated concrete (AAC) [8].

Industrial processes generate substantial quantities of by-products, such as 300 kg of blast furnace slag (BFS) per ton of pig iron produced [9]. Moreover, the global biofuel industry produces around 320 million tons of by-products, including fly ash (FA) [9,10,11]. Improper handling of these by-products can lead to serious environmental risks. Consequently, extensive research has focused on their reuse, with AAC production offering a key pathway for valorizing these materials [12].

In AAC production, industrial by-products such as ground granulated blast furnace slag (GGBFS) react with alkaline activators to form a polymeric binder, which combines with coarse and fine aggregates to create the final product [13,14]. These geopolymer binders can partially or fully replace OPC, reducing both energy consumption and CO_2_ emissions while facilitating the secondary utilization of industrial waste—a critical step toward environmental sustainability [15].

Despite AAC’s potential as a sustainable OPCC alternative, accurately predicting its mechanical properties remains a challenge. Variability in mix design, raw material sources, and curing conditions introduce significant uncertainty, complicating the optimization of AAC formulations for specific applications [16]. Traditional predictive methods, while useful, often fail to fully capture these complexities. In recent years, however, advances in computational techniques have opened new avenues for addressing these challenges. Here, machine learning (ML), a powerful tool within artificial intelligence, emerges as a promising solution. By leveraging its ability to analyze large datasets, ML models can effectively capture the complex relationships between mix parameters and AAC properties, leading to improved prediction accuracy.

Several recent studies have demonstrated the efficacy of ML in this domain. For instance, Nguyen et al. [17] utilized a deep residual network (ResNet) trained on 339 samples of FA-based geopolymer concrete, achieving a remarkable correlation coefficient (R) of 0.9927 in predicting compressive strength. Ahmad et al. [18] employed a bagging regression (BR) model to forecast compressive strength in fly ash-based geopolymer concrete, attaining a determination coefficient (R^2^) of 0.97. Similarly, Zhang et al. [19] applied a gradient boosting regressor (GBR) to alkali-activated materials, reaching an R^2^ of 0.944. In another study, Tanyildizi utilized a deep long short-term memory (LSTM) model to predict various geopolymer properties, achieving accuracies exceeding 99% across multiple metrics [20]. These results underscore the potential of ML models to provide accurate predictions across different AAC formulations and scales from the microstructural to the macroscopic level.

However, while ML models excel in prediction, they often lack transparency regarding the contribution of individual variables to the model’s output. To address this, SHapely Additive exPlanation (SHAP) has gained traction for interpreting model results [21]. SHAP leverages game theory principles to assess the contribution of each variable (or “player”) to the overall outcome, providing a clearer understanding of the factors influencing AAC performance.

Building on our previous work [22], we adopted a comprehensive database compiled from 111 AAC studies, consisting of 3390 data points, each representing a unique AAC mix design with compressive strength as the target output. To assess the influence of input variables, we devised and compared four distinct input variable schemes, ultimately selecting the optimal one for further analysis. We then evaluated the performance of four machine learning models—random forest (RF), adaptive boosting (AdaBoost), gradient boosting regression trees (GBRTs), and extreme gradient boosting (XGBoost)—across these schemes. Notably, the XGBoost model demonstrated higher predictive accuracy, consistently outperforming the other algorithms.

With XGBoost identified as the top-performing model, we sought to further enhance its accuracy by applying four advanced optimization techniques, namely the Gray Wolf Optimizer (GWO), Whale Optimization Algorithm (WOA), beetle antennae search (BAS), and Bayesian optimization (BO). Among these, the GWO yielded the highest predictive performance, achieving an R^2^ value of 0.94 across the entire dataset. The WOA followed closely, with a maximum R^2^ of 0.93, while BAS and BO each reached an R^2^ of 0.92. Based on these results, we selected the GWO-optimized XGBoost model as the final predictive model.

To further illuminate the model’s inner workings and ensure it did not function as a “black box”, we employed SHAP for interpretability analysis. SHAP enabled us to quantify the importance of each input variable, offering insights into their individual contributions to compressive strength predictions. In addition, we visualized these insights through partial dependence plots (PDPs) and individual conditional expectation (ICE) plots, which provided a more granular understanding of how specific variables influence the model’s predictions. This analysis not only enhanced transparency but also deepened our understanding of AAC behavior, reinforcing the model’s reliability in practical applications.

## 2. Research Importance and Workflow

Extensive research has explored the effects of supplementary cementitious materials, such as FA and GGBFS, on concrete properties [23,24,25]. Within this context, ML has emerged as a potent tool for analyzing data and predicting the compressive strength of AAC [26,27,28,29,30]. A key aspect of ML model development is hyperparameter tuning, which is a critical step in enhancing model prediction accuracy. Several studies have investigated various optimization methods for this purpose. For instance, Parhi et al. applied the Spotted Hyena Optimization algorithm to optimize hyperparameters for models such as LASSO regressors, support vector regressors, random forest, bagging regressors, AdaBoost, and XGBoost, ultimately achieving highly accurate predictions [31]. Similarly, Zhang et al. utilized genetic algorithms and 10-fold cross-validation to fine-tune backpropagation neural networks and support vector machines, resulting in an improved prediction accuracy for the final setting time and uniaxial compressive strength [32].

These studies consistently show that optimized ML models—through hyperparameter tuning—can significantly enhance performance. Equally important, however, is the careful selection of input variables, as they directly impact the model’s ability to capture critical patterns in the data. Together, hyperparameter tuning and input variable selection are fundamental to building robust and reliable AI models, particularly in challenging fields like AAC strength prediction.

**Figure 1:**
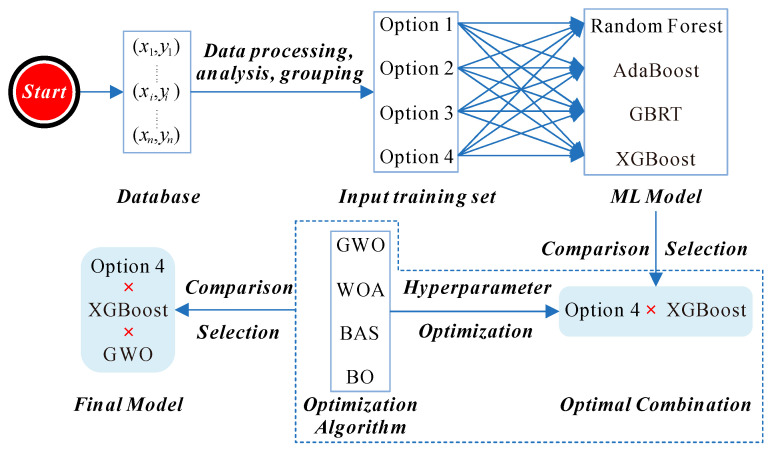
Workflow of this study.

Building on these insights, our study focused on improving the prediction of AAC compressive strength by developing an ML model optimized through advanced hyperparameter tuning algorithms. Unlike prior research, we also placed special emphasis on the thoughtful selection of input variables, which served as the foundation for constructing a highly accurate model. The workflow for this model development process is depicted in Figure 1. In the figure, Options 1 to 4 represent the four input variable schemes.

Once the model is built, a comprehensive understanding of the influence of each input variable becomes crucial. An increasing number of studies have turned to model interpretation techniques to achieve this. SHAP, a widely used tool for interpreting ML models, has been applied in numerous studies to analyze the individual contributions of input variables [33,34]. In this study, we employed both the built-in analysis functions of the XGBoost algorithm and the SHAP library to interpret the final optimized model. This dual approach provides deeper insights into the model’s decision-making process and its practical implications for AAC production.

The integration of ML into AAC research, combined with thoughtful input variable selection, rigorous hyperparameter tuning, and advanced model interpretation, has proven increasingly vital for improving predictive accuracy and transparency. By leveraging these techniques, our study not only enhanced the precision of AAC compressive strength predictions but also demystified the model’s decision-making process, turning it into a reliable and interpretable tool. Thus, through the careful alignment of these methods, we have developed a robust and efficient ML model that offers greater confidence in predicting AAC compressive strength, ultimately providing valuable insights for optimizing mix designs and advancing sustainable construction practices.

## 3. An Overview of ML Models and Optimization Algorithms

This section outlines the machine learning models and optimization algorithms employed in this study. Due to space limitations, we will focus on the most essential concepts. Readers interested in a more detailed explanation are encouraged to consult the relevant references.

### 3.1. Machine Learning Models

#### 3.1.1. Random Forest (RF)

RF is a widely used ensemble learning technique. It falls under the category of bagging methods, where the underlying structure consists of multiple decision trees [35]. Ensemble learning, in essence, generates a collection of weak learners by processing training data and combines them in a specific way to form a more powerful robust learner. The two main types of ensemble learning are boosting and bagging. In bagging, weak learners are generated independently and in parallel, while in boosting, the learners are interconnected.

RF is a bagging-based model that uses decision trees to create its ensemble of learners [36]. In this approach, each decision tree is structured as a series of nodes, where each node represents an attribute and splits the data step-by-step in an attempt to approximate the target output. When used for regression tasks, the RF model predicts the outcome by averaging the outputs of all individual trees, thus improving accuracy and reducing variance.

To enhance predictive accuracy, tuning RF hyperparameters is essential. The “n_estimators” parameter determines the number of trees in the forest. While increasing the number of trees generally improves model stability and accuracy, it also increases computational demands. The “max_depth” parameter controls the depth of each tree, with deeper trees capturing more complex patterns but potentially risking overfitting. Conversely, setting a shallower depth prevents overfitting by simplifying the model. The “max_leaf_nodes” parameter limits the number of terminal nodes, which can also help prevent overfitting by reducing the model’s complexity.

Other important parameters include “min_samples_leaf” and “min_samples_split”, which specify the minimum number of samples needed at a leaf node and for splitting a node, respectively, helping to control the model’s complexity. The “bootstrap” parameter determines whether bootstrapping (sampling with replacement) is used when constructing trees, which can improve the model’s generalizability. The “random_state” parameter sets a seed for random number generation, ensuring the reproducibility of results. Lastly, “max_features” defines the maximum number of features considered for each split, promoting model diversity and reducing the impact of correlated features.

#### 3.1.2. Adaptive Boosting (AdaBoost)

**Figure 2:**
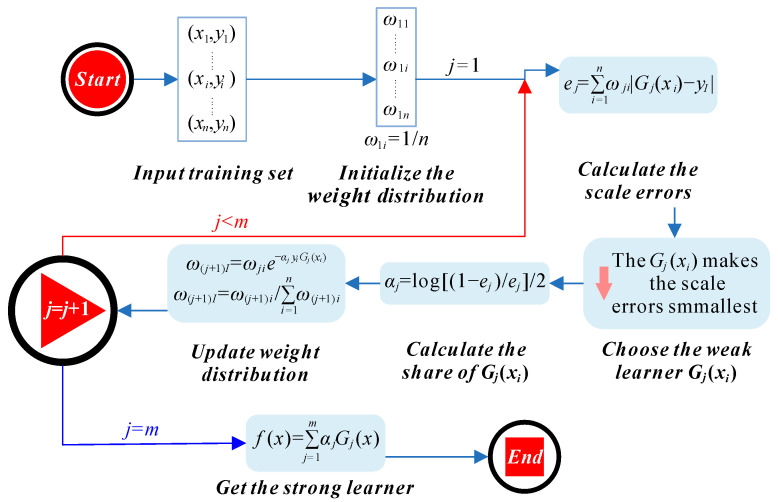
The principle of AdaBoost.

AdaBoost is a key member of the boosting family of ensemble learning methods. The core principle of boosting involves generating a series of weak learners, each trained with adjusted weights based on the performance of previous learners. Initially, all training samples are given equal weight. As the boosting process progresses, the algorithm increases the weight of misclassified samples, directing subsequent learners to focus on these harder cases. This iterative approach continues until the specified number of weak learners is achieved, with all weak learners combined to form a robust strong learner for prediction [37]. Figure 2 illustrates the AdaBoost principle, highlighting how each weak learner is integrated into the final model to enhance overall predictive performance [38].

When fine-tuning the hyperparameters to optimize the performance of the AdaBoost model, several parameters play roles similar to those in RF. These include “n_estimators”, which specifies the number of weak learners, and “max_depth”, “max_leaf_nodes”, “min_samples_leaf”, “min_samples_split”, and “random_state”, which help control the complexity and reproducibility of the model.

In addition to these, AdaBoost introduces the “learning_rate” parameter, which is critical in determining the weight of each weak learner in the final model. A lower learning rate necessitates the use of more weak learners to achieve optimal performance, while a higher learning rate speeds up the learning process but may increase the risk of overfitting. Thus, careful adjustment of the learning rate is essential for balancing model performance and generalization.

#### 3.1.3. Gradient Boosting Regression Tree (GBRT)

GBRT is a powerful boosting algorithm used for regression tasks, which combines the strengths of regression trees (RTs) with gradient boosting (GB). In GBRT, the core idea involves building a sequence of regression trees, where each successive tree aims to correct the errors made by the previous ones. This iterative approach refines the model by minimizing a specified loss function, making GBRT particularly effective for complex regression problems.

**Figure 3:**
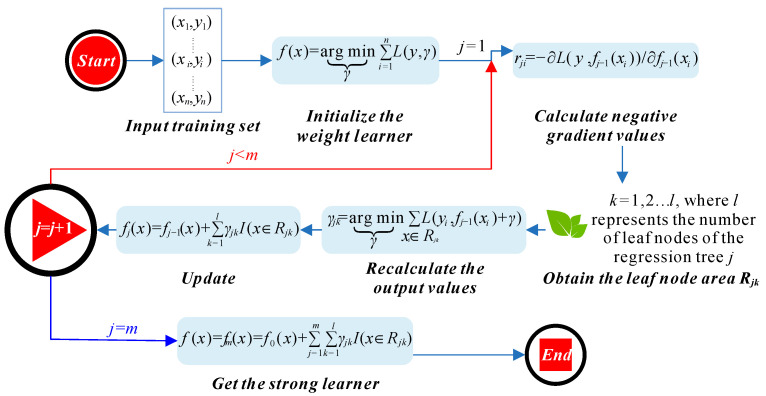
The principle of GBRT.

GBRT builds on the principles of gradient boosting, a method that optimizes the model by fitting new trees to the residual errors of the existing ensemble. Unlike AdaBoost, which adjusts weights based on misclassified samples, GBRT focuses on minimizing the loss function directly through each iteration, refining the predictions incrementally. This makes GBRT well-suited for capturing intricate patterns in the data and improving the overall predictive performance [39]. The principle of GBRT, including its iterative nature and loss function minimization, is depicted in Figure 3.

In GBRT, the hyperparameters “n_estimators”, “max_depth”, “learning_rate”, “min_samples_leaf”, “min_samples_split”, and “random_state” serve similar functions to those in RF and AdaBoost. Specifically, they can be defined as follows:

- n_estimators: specifies the number of trees in the ensemble.
- max_depth: defines the maximum depth of each tree, controlling model complexity.
- learning_rate: adjusts the contribution of each tree to the final model, with lower rates requiring more trees to achieve good performance.
- min_samples_leaf: sets the minimum number of samples required at a leaf node, preventing overfitting.
- min_samples_split: determines the minimum number of samples needed to split an internal node, regulating tree complexity.
- random_state: ensures reproducibility by controlling the random seed used in model training.

These hyperparameters collectively influence the model’s accuracy, stability, and generalization capabilities.

#### 3.1.4. Extreme Gradient Boosting (XGBoost)

XGBoost, a prominent member of the boosting family in integrated learning, builds on the foundational principles of gradient boosting but incorporates additional advancements that enhance its performance and flexibility. While GBRT typically fits the first-order derivative of the loss function, XGBoost improves upon this by utilizing the second-order Taylor expansion of the loss function. This allows XGBoost to leverage both first- and second-order derivatives, resulting in more refined model fitting and improved predictive accuracy [40].

The core principle of XGBoost involves optimizing an objective function that balances model accuracy with complexity. For a given training set *U* = {(*x*_1_, *y*_1_), (*x*_2_, *y*_2_), …, (*x_i_*, *y_i_*), …, (*x_n_*, *y_n_*)}, where n represents the number of samples, *x*_i_ denotes the input features, and *y*_i_ represents the true output values, the goal is to minimize the loss function *L*. The objective function in XGBoost incorporates both the loss function and a regularization term to penalize model complexity.

(1) $$\mathrm{Objective}\;\mathrm{Function}:\;\;\;obj(\theta )=\sum_{i=1}^{n}L(y_{i},\hat{y_{i}})+\sum_{j=1}^{m}\Omega (f_{j}),$$

where *ŷ_i_* is the predicted value of the tree model for the *i*th sample; *y_i_* is the actual value of the *i*th sample; *j* is the number of trees, *j* = 1, 2, …, *m*; *m* is the total number of trees; *f_j_* is the model of the *j*th tree; and the regularization term is $\Omega (f)=\gamma T+\frac{1}{2}\lambda \sum_{k=1}^{T}\omega_{k}^{2}$, in which *T* is the total number of leaf nodes in a tree; *ω_k_* is the predicted value of the *k*th leaf node; and *γ* and *λ* are the factors to control the weighting of these two components.

After a series of deductions and simplifications, the objective function for XGBoost can be expressed as

(2) $$obj^{\left(t\right)}=-\frac{1}{2}\sum_{k=1}^{T}\left(\frac{G_{k}^{2}}{H_{k}+\lambda }\right)+\gamma T+c,$$

where $G_{k}=\sum_{i\in I_{k}}g_{i}$ and $H_{k}=\sum_{i\in I_{k}}h_{i}$, in which $g_{i}=\frac{\partial L(y_{i},{\hat{y_{i}}}^{\left(t-1\right)})}{\partial {\hat{y_{i}}}^{\left(t-1\right)}}$, $h_{i}=\frac{\partial^{2}L(y_{i},{\hat{y_{i}}}^{\left(t-1\right)})}{\partial {\left({\hat{y_{i}}}^{\left(t-1\right)}\right)}^{2}}$, and *c* is a constant.

This formula, also known as the scoring function, measures the merit of a tree in terms of its loss function and the complexity of the tree.

To find the optimal model, XGBoost uses a greedy algorithm to construct trees that minimize the objective function. The decision on how to split nodes is based on the gain, which is calculated as follows:

(3) $$\begin{array}{l} Gain=obj_{L+R}-\left(obj_{L}+obj_{R}\right)\\ =\frac{1}{2}\left[\frac{G_{L}^{2}}{H_{L}+\lambda }+\frac{G_{R}^{2}}{H_{R}+\lambda }-\frac{{\left(G_{L}+G_{R}\right)}^{2}}{H_{L}+H_{R}+\lambda }\right]-\gamma \end{array}$$

For fine-tuning XGBoost, several hyperparameters are crucial, including the following:

- n_estimators: the number of boosting rounds or trees to build, analogous to GBRT and random forest.
- max_depth: the maximum depth of each tree, balancing model complexity and overfitting risk.
- learning_rate: controls the contribution of each tree to the final model, with a lower rate requiring more trees to achieve optimal performance.
- random_state: ensures the reproducibility of results by setting a random seed.

The above parameters in XGBoost serve functions analogous to those in GBRT. However, XGBoost introduces additional hyperparameters that offer greater control over the model’s performance and robustness as follows:

- colsample_bytree: specifies the fraction of features to be used for each tree, which introduces randomness and reduces overfitting.
- subsample: defines the proportion of samples used to build each tree, further enhancing model robustness and mitigating overfitting.
- gamma: determines the minimum loss reduction required for a further split on a leaf node, thereby controlling overfitting by setting a threshold for split improvement.

These parameters collectively enhance XGBoost’s ability to handle complex datasets and improve its predictive performance by refining model fitting and robustness.

### 3.2. Optimization Algorithms

#### 3.2.1. Gray Wolf Optimizer (GWO)

The GWO algorithm is a meta-heuristic inspired by the social hierarchy and predatory behavior of gray wolves. This algorithm mimics the natural pack dynamics of wolves to solve complex optimization problems.

In the gray wolf pack, the social hierarchy is organized into four distinct roles, namely *α*, *β*, *δ*, and *ω*. The *α* wolves hold the highest leadership and decision-making power, followed by *β* and *δ*, with decreasing authority. The *ω* wolves, being at the bottom of the hierarchy, lack leadership influence. In the GWO algorithm, the *α*, *β*, and *δ* wolves are responsible for guiding the search process by locating and encircling the prey, while the *ω* wolves follow and contribute to the collective pursuit of the target.

The principle of the GWO algorithm involves simulating these hierarchical behaviors to optimize solutions. The algorithm employs the *α*, *β*, and *δ* wolves to lead the search, adjusting positions based on prey location and iteratively refining the search strategy through encirclement and attack behaviors. This mimics the wolves’ natural hunting strategies and helps in efficiently exploring the solution space.

**Figure 4:**
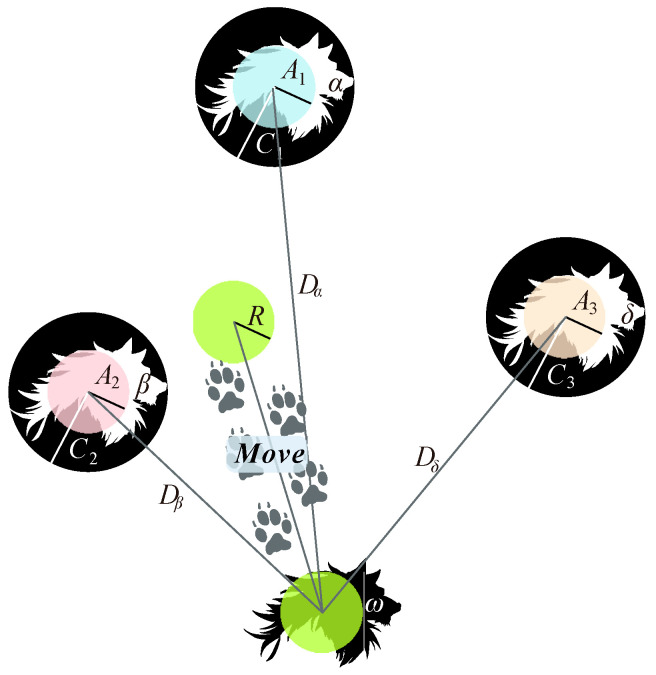
The principle of the GWO algorithm.

Figure 4 illustrates the principle of the GWO algorithm, showcasing how the wolves’ roles and behaviors are translated into optimization processes.

The GWO algorithm proceeds as follows [41]:

1. Surrounding

This process can be expressed by the following equations:

(4) $$\vec{X}(t+1)=\vec{X^{*}}(t)-\vec{A}\cdot \vec{D},$$

where $\vec{D}=\left|\vec{C}\cdot \vec{X^{*}}(t)-\vec{X}(t)\right|$; $\vec{A}=2\vec{a}\cdot \vec{r_{1}}-\vec{a}$; and $\vec{C}=2\vec{r_{2}}$. $\vec{r_{1}}$, $\vec{r_{2}}$ represent random numbers chosen from 0 to 1; $\vec{a}$ decreases linearly from 2 to 0 over the course of the iterations; and $\vec{X^{*}}(t)$ represents the best performing position vector.

2. Hunting

Using the information from *α*, *β*, and *δ*, the following equation was used to update the position of each individual gray wolf:

(5) $$\vec{D_{\alpha }}=\left|\vec{C_{1}}\cdot \vec{X_{\alpha }}-\vec{X}\right|,\vec{D_{\beta }}=\left|\vec{C_{2}}\cdot \vec{X_{\beta }}-\vec{X}\right|,\vec{D_{\delta }}=\left|\vec{C_{3}}\cdot \vec{X_{\delta }}-\vec{X}\right|,$$

(6) $$\vec{X_{1}}=\vec{X_{\alpha }}-\vec{A_{1}}\cdot \vec{D_{\alpha }},\vec{X_{2}}=\vec{X_{\beta }}-\vec{A_{2}}\cdot \vec{D_{\beta }},\vec{X_{3}}=\vec{X_{\delta }}-\vec{A_{3}}\cdot \vec{D_{\delta }},$$

(7) $$\vec{X}(t+1)=\frac{\vec{X_{1}}+\vec{X_{2}}+\vec{X_{3}}}{3},$$

where $\vec{X_{\alpha }}$, $\vec{X_{\beta }}$, and $\vec{X_{\delta }}$ denote the position vectors of *α*, *β*, and *δ* in the current population, respectively; $\vec{X}$ denotes the position vector of gray wolves; $\vec{D_{\alpha }}$, $\vec{D_{\beta }}$, and $\vec{D_{\delta }}$ denote the distance between the current candidate gray wolves and *α*, *β*, and *δ*, respectively; when |$\vec{A}$| > 1, gray wolves try to spread out among regions and search for prey; when |$\vec{A}$| < 1, the gray wolves will concentrate on searching for prey in a certain area.

3. Attacking

This process is mainly controlled by $\vec{A}=2\vec{a}\cdot \vec{r_{1}}-\vec{a}$. The linear decrease in a from 2 to 0 also results in a change in the *A* value. When |$\vec{A}$| ≤ 1, the wolves will move closer to their prey; when 1 <|$\vec{A}$| ≤ 2, the wolves will scatter away from their prey, causing the algorithm to fall into a local optimum.

#### 3.2.2. Whale Optimization Algorithm (WOA)

The WOA is also a meta-heuristic algorithm inspired by the hunting behavior of humpback whales, particularly their bubble-net feeding technique. The WOA is designed to solve optimization problems by simulating the way these whales encircle and capture their prey. The algorithm operates through the following key steps [42]:

1. Encircling prey

In this phase, the WOA models the behavior of whales encircling their prey. This is achieved using the following equations:

(8) $$\vec{X}(t+1)=\vec{X^{*}}(t)-\vec{A}\cdot \vec{D}=\vec{X^{*}}(t)-\left(2\vec{a}\cdot \vec{r}-\vec{a}\right)\cdot \left|\vec{C}\cdot \vec{X^{*}}(t)-\vec{X}(t)\right|,$$

where $\vec{C}=2\vec{r}$. $\vec{r}$ represents a random number chosen from 0 to 1 and $\vec{a}$ decreases linearly from 2 to 0 over the course of the iterations.

2. Bubble-net attacking method

Humpback whales use bubble-net feeding to trap their prey. This behavior is simulated in two distinct ways as follows:

- Shrinkage envelope mechanism

This mechanism simulates the whale’s ability to create a shrinking bubble net around the prey, reducing the value of $\vec{a}$ in $\vec{A}=2\vec{a}\cdot \vec{r}-\vec{a}$, where the range of fluctuations of $\vec{A}$ also diminishes as the value of $\vec{a}$ decreases, allowing the whale to focus more closely on the prey.

- Spiral update position

This mechanism simulates the spiral movement of the whale around the prey. It first calculates the distance between the whale at (*X*, *Y*) and the prey at (X*, Y*). A spiral equation is then created between the positions of the whale and the prey, which is given below.

(9) $$\vec{X}(t+1)=\vec{D^{\prime }}\cdot e^{bl}\cdot \cos(2\pi l)+\vec{X^{*}}(t),$$

where $\vec{D^{\prime }}=\left|\vec{X^{*}}(t)-\vec{X}(t)\right|$.

Both mechanisms above are used with equal probability (50%) to simulate the hunting behavior effectively.

3. Searching for prey

When searching for prey, humpback whales perform random searches based on the positions of other whales. This is akin to the encircling prey phase (Equation (8)), but $\vec{X^{*}}(t)$ turns into $\vec{X_{rand}}$, which represents an arbitrary location trajectory, and $\vec{D}=\left|\vec{C}\cdot \vec{X_{rand}}-\vec{X}\right|$.

This simulation of whale behavior allows the WOA to effectively explore the solution space and find optimal solutions by combining exploration and exploitation strategies.

#### 3.2.3. Beetle Antennae Search Algorithm (BAS)

The BAS algorithm is a single-unit search algorithm inspired by the foraging behavior of beetles in nature. The algorithm mimics how these beetles use their antennae to locate food sources.

The BAS algorithm proceeds as follows [43]:

1. Determining the position of the left and right antennae

The position of the left and right antennae of the Tenebrae can be expressed as

(10) $$\left\{\begin{array}{c} \vec{X_{r}}=\vec{X}+l\cdot \vec{d}\\ \vec{X_{l}}=\vec{X}-l\cdot \vec{d} \end{array}\right.,$$

where *l* denotes the distance between the center of mass of the beetle and the antennae; $\vec{d}$ denotes a random unit vector, $\vec{d}=\frac{rand\left(D,1\right)}{\left\|rand\left(D,1\right)\right\|}$; and $\vec{X}$ represents the current position of the beetle.

2. Determining the next position of the beetle

To simulate how the beetle makes directional judgments based on the difference in odor concentration detected by its antennae, the following equation is used:

(11) $$\vec{X}(t+1)=\vec{X}(t)+\delta_{t}\cdot \vec{d}\cdot sign\left[f\left(\vec{X_{r}}\right)-f\left(\vec{X_{l}}\right)\right],$$

where *f*(*x*) represents the fitness function; $\delta_{t}$ represents the step size at the *t*th iteration, $\delta_{t+1}=\delta_{t}\cdot eta$; and *sign*(*x*) denotes the sign function, which determines the direction of movement based on the fitness evaluation. Note that the sign function is commonly used in optimization algorithms to determine the direction of changes or movements based on certain criteria. The function is defined as follows: sign(*x*) = 1 if *x* > 0; sign(*x*) = −1 if *x* < 0; and sign(*x*) = 0 if *x* = 0. In the context of the BAS algorithm, the sign function is used to guide the search process based on the fitness evaluation of different positions. For example, if a position is found to be better (higher fitness) than the current one, the sign function will indicate a positive direction (1), suggesting that the search should move toward this position. Conversely, if the position is worse (lower fitness), the sign function will indicate a negative direction (−1), suggesting movement away from that position.

#### 3.2.4. Bayesian Optimization (BO)

The BO algorithm applies Bayes’ theorem to guide the optimization process [44]. The fundamental equation is

(12) $$p\left(f\left|D_{i}\right.\right)=\frac{p\left(D_{i}\left|f\right.\right)p\left(f\right)}{p\left(D_{i}\right)},$$

where *f* denotes the unknown objective function; *D_i_* = {(*x*_1_, *y*_1_), (*x*_2_, *y*_2_), …, (*x_i_*, *y_i_*), …, (*x_n_*, *y_n_*)} denotes the observed set, *x_i_* denotes the decision vector, and *y_i_* = *f*(*x_i_*) denotes the observation error; *p*(*D_i_*|*f*) denotes the likelihood distribution of *y*; *p*(*f*) denotes a prior probability distribution; *p*(*D_i_*) denotes the likelihood distribution of marginal *f*; and *p*(*f*|*D_i_*) denotes the posterior probability distribution of *f*. The posterior probability distribution describes the confidence level of the unknown objective function after the prior probability distribution has been corrected by the observed dataset.

In Bayesian optimization, a Gaussian process (GP) is often used to model the distribution of the objective function. The GP is characterized by a mean function *μ* and a covariance kernel function *K*, and is denoted as *f* ~ *GP*(*μ*, *K*).

Once the posterior distribution *p*(*f*|*D_i_*) is updated, the algorithm identifies the next hyperparameters to evaluate using an acquisition function, such as the upper confidence bound (UCB) method. This approach iterates until the optimization process converges, optimizing the objective function effectively with each iteration.

#### 3.2.5. Comments on the above Optimization Algorithms

The GWO algorithm excels in simulating natural predatory behaviors to explore and exploit the search space, offering a robust mechanism for optimization. However, it may sometimes suffer from convergence issues, particularly in highly complex or multimodal optimization landscapes.

The WOA provides a flexible approach with mechanisms such as bubble-net attacks and spiral updates, enhancing its ability to navigate complex spaces. Yet, the WOA can be sensitive to parameter settings and may require careful tuning to balance exploration and exploitation effectively.

The BAS algorithm offers a unique approach by simulating the directional judgments of antennae to refine search strategies. While BAS can provide precise local search capabilities, its performance can be heavily influenced by the initial conditions and the specific problem domain.

The BO algorithm performs well in handling expensive evaluation functions and provides a probabilistic framework for exploring the search space. However, BO can be computationally intensive and may struggle with very high-dimensional problems or scenarios where the function evaluations are highly noisy.

In summary, while the GWO, WOA, BAS, and BO each bring distinct methodologies to optimization, they also come with trade-offs. In this study, we will provide a detailed comparative analysis of these four optimization algorithms, evaluating their effectiveness in the context of predicting AAC’s compressive strength.

## 4. Data Preparation and Input Variable Selection

This section provides a brief description of the database used for the model (including its characteristics) and how the input variable sets were prepared.

### 4.1. Database Construction

This paper used a comprehensive experimental database compiled from 1756 individual AAC mixtures reported in 111 studies [22]. The database covers a wide array of manufacturing conditions, ranging from macroscopic variables like coarse and fine aggregate volumes and water-to-ash ratios to microscopic chemical compositions. It also includes detailed raw material configurations and the energy changes during the curing process. Indeed, this rich diversity of data provides a foundation for robust ML modeling.

To standardize and integrate the data from these various experiments, certain adjustments and conversions were performed. For instance, all ionic compounds involved in the reaction were converted into their equimolar oxide forms. This step ensures consistency across the dataset, facilitating accurate model training. Additionally, some input variables were derived by combining existing data. For example, the relative modulus (RM), formulated using the masses of calcium oxide (CaO), sodium oxide (Na_2_O), silica (SiO_2_), alumina (Al_2_O_3_), and iron oxide (Fe_2_O_3_), is defined as follows [21]:

(13) $$\mathrm{RM}=\frac{{\mathrm{CaO}+\mathrm{Na}}_{2}O}{{\mathrm{SiO}}_{2}{+\mathrm{Al}}_{2}O_{3}{+\mathrm{Fe}}_{2}O_{3}}$$

In this equation, the oxides in the denominator reflect the alkalinity of the feedstock, while those in the numerator represent the potential to form a polymeric or hydrated product, essential in AAC’s final structure.

To further integrate the data, additional transformations were applied. For example, the ratio of sodium silicate (SS) to sodium hydroxide (SH) by mass was calculated, reflecting its influence on the mechanical performance of AAC. Aggregate properties were converted from mass to volume [45], assuming a relative density of 2.6, ensuring more precise representation in the model.

Moreover, two key input variables were introduced to capture the relationship between the water solution and gel content. The first (L/S) represents the ratio of the sum of SS, SH, and added water to the total weight of the gelling material; the second (W/S) includes superplasticizer content alongside SS, SH, and added water divided by the total weight of the cementitious material.

Curing regimes, which have a significant impact on AAC’s compressive strength, were also factored into the model. To quantify the effect of the curing temperature, the total energy consumed per unit weight of AAC during the curing process was calculated using the following equation, as proposed in [46]:

(14) $$Energy_{curing}=m\cdot c\cdot (T_{curing}-25)\cdot t;T_{curing}>25\;{}^{\circ}C,$$

where *m* represents the bulk density of ACC (assumed to be 2400 kg/m^3^); *c* represents the specific heat capacity of AAC (700 J/kg °C); and *T_curing_* and *t_curing_* represent, respectively, the curing temperature (°C) and time (days). For ambient curing conditions, where temperatures remain below 25 °C, energy consumption was considered to be zero.

Finally, the shape of the test specimens, recorded as discrete data in the database, was also standardized. Rectangular prisms were assigned a value of 1 and cylinders a value of 2. This discrete classification enabled us to incorporate geometrical variations into the model.

To offer a clearer view of the input parameters utilized in this study, we have summarized the key variables in Table 1. These variables form the basis of the subsequent analysis, providing the foundation for our machine learning-driven approach to AAC strength prediction.

To clarify the scope of each parameter in this dataset, it is apparent that the values comprehensively encompass the typical range observed in practical AAC applications. This broad coverage ensures that the dataset captures the extreme and intermediate cases, offering a realistic reflection of actual manufacturing scenarios. The regression analysis used to predict AAC compressive strength leverages the relationships between the input variables and the output target, providing insights into how each parameter influences the model’s predictions.

In machine learning-based models for AAC strength prediction, the model’s effectiveness hinges on the quality and representativeness of the input variables. As long as the input variables fall within a reasonable range—one that reflects practical boundaries—the model should be able to generate accurate and reliable predictions. This makes the dataset particularly valuable, as it not only covers a wide range of experimental conditions but also ensures that the model is robust across varying AAC formulations.

Therefore, the diversity and comprehensiveness of this dataset form a reliable basis for improving the model’s generalizability and predictive accuracy. It equips the model to handle real-world variability in AAC formulations, making it well-suited for machine learning applications.

### 4.2. Data Exploration and Insights

In this section, we provide a detailed analysis of the dataset used for modeling AAC compressive strength, focusing on the distribution characteristics of input variables and the rationale behind their selection.

**Figure 5:**
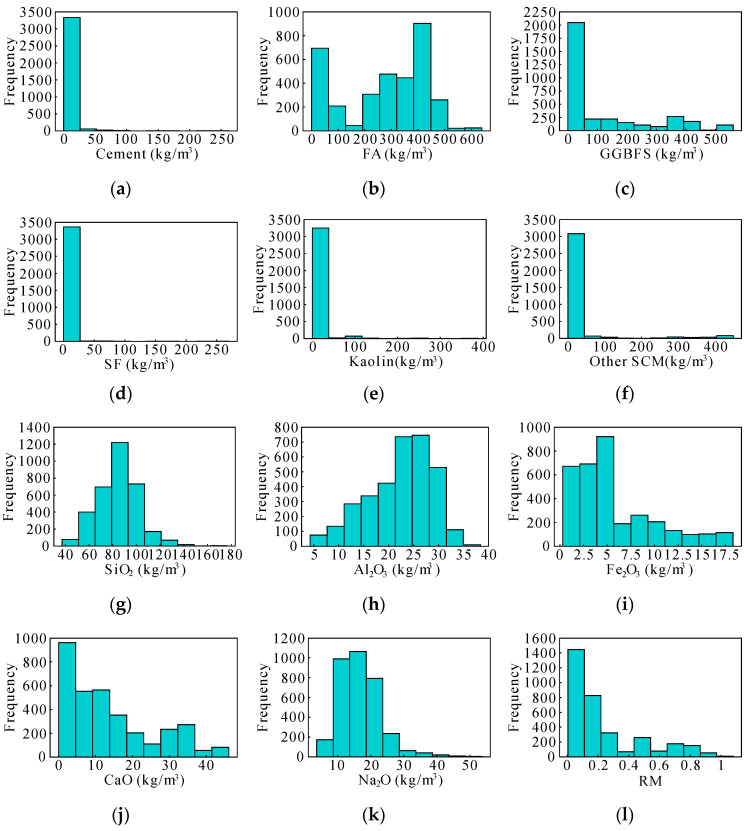

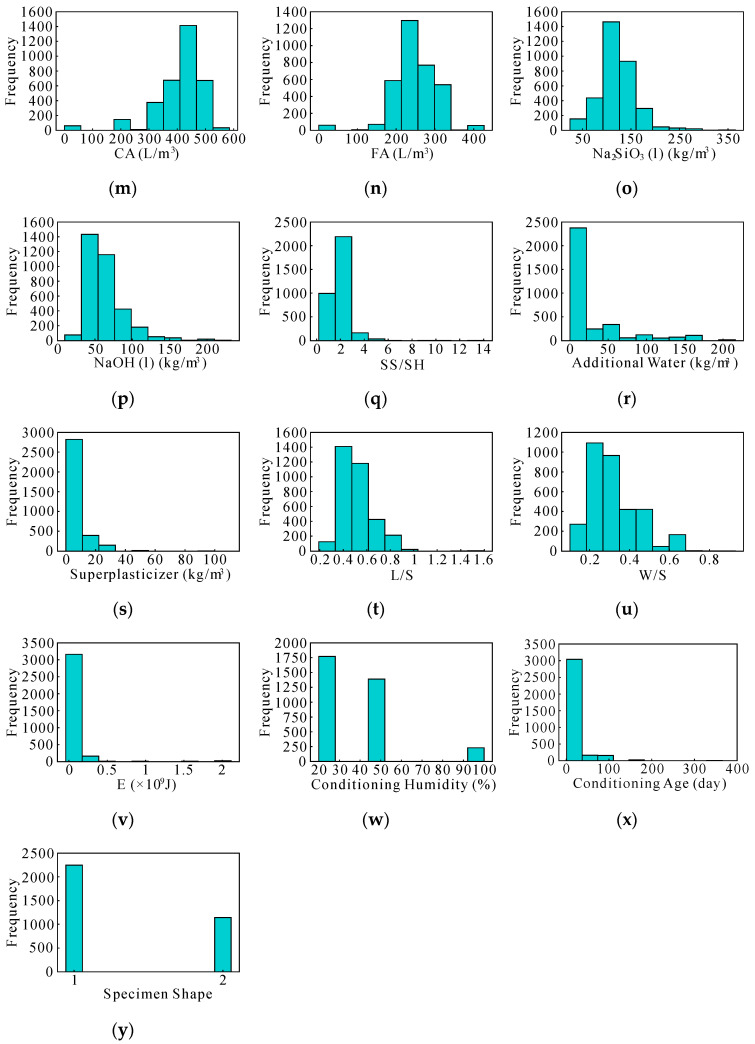
Sample distribution of each input variable: (**a**) cement (kg/m^3^); (**b**) FA (kg/m^3^); (**c**) GGBFS (kg/m^3^); (**d**) SF (kg/m^3^); (**e**) kaolin (kg/m^3^); (**f**) other SCM (kg/m^3^); (**g**) SiO_2_ (kg/m^3^); (**h**) Al_2_O_3_ (kg/m^3^); (**i**) Fe_2_O_3_ (kg/m^3^); (**j**) CaO (kg/m^3^); (**k**) Na_2_O (kg/m^3^); (**l**) RM; (**m**) CA (L/m^3^); (**n**) FA (L/m^3^); (**o**) Na_2_SiO_3_ (l) (kg/m^3^); (**p**) NaOH (l) (kg/m^3^); (**q**) SS/SH; (**r**) additional water (kg/m^3^); (**s**) superplasticizer (kg/m^3^); (**t**) L/S; (**u**) W/S; (**v**) E (J); (**w**) curing humidity (%); (**x**) curing age (day); and (**y**) specimen shape.

To gain a preliminary understanding of the data distribution, histograms were constructed for each input variable, as shown in Figure 5. This visual representation allowed for an intuitive grasp of the variability present within the dataset. Upon examination, the distributions of most input variables appeared reasonable, although certain outliers were observed. For example, the first six variables correspond to different types of cementitious materials. Due to variations in mix proportions, some of these materials, especially those present in minimal quantities, exhibited a significant number of zero values across the samples. This led to an uneven distribution. Similarly, while curing humidity and curing age typically fall within standard ranges, certain samples—such as those with extended curing ages of up to 365 days—exhibited broader distributions. Despite such variations, these cases reflect realistic experimental conditions and are therefore considered valid.

**Figure 6:**
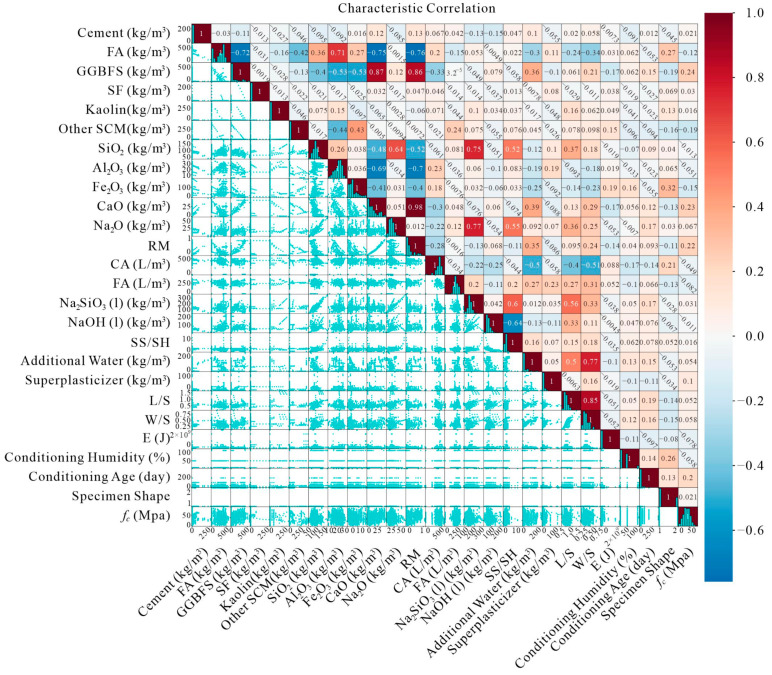
Variable correlation diagram.

A key challenge in constructing machine learning models is addressing the potential redundancy caused by highly correlated input variables. Including variables with strong correlations, such as SiO_2_ and Na_2_O, may lead to a model that redundantly references overlapping data features, thereby impairing its predictive power. To mitigate this, we performed a thorough correlation analysis, visualizing the relationships between input variables through scatter plots, bar charts, and heat maps, as depicted in Figure 6. The upper-right quadrant of Figure 6 highlights significant correlations, which are further elucidated by the heatmap, while the lower-left quadrant illustrates both positive and negative correlations among the variables. These insights are crucial for ensuring that input variable combinations do not constrain the model by imposing overly rigid relationships.

Given the importance of carefully selecting input variables, we prioritized eliminating combinations with excessive correlations to avoid redundant influences on AAC compressive strength predictions. This comprehensive approach ensures that the model retains flexibility and generalizability across varying AAC formulations.

Before finalizing the dataset for modeling, we conducted an outlier detection process to identify any anomalous data points. Outliers, defined as data that deviate significantly from the general dataset, can arise from systematic or random experimental errors. While methods such as the tails of normal distribution, Thompson Tau, Box plots, T-test, Dixon criteria, and Grubbs test are commonly used for outlier detection, real-world datasets often contain inherent variability and errors [47,48,49]. As seen in Figure 6, no significant outliers were identified in our dataset. Therefore, all data points were retained to reflect the inherent variability present in practical applications.

Unlike many studies where input variables are preselected at the project’s outset [50,51], our approach places greater emphasis on the rigorous selection of variables. Figure 5 and Figure 6 not only illustrate the data distribution but also highlight the correlations, providing a solid empirical basis for choosing the most informative and non-redundant input variables. This careful selection process forms the foundation for developing robust and interpretable models for AAC compressive strength prediction.

### 4.3. Selection of Input Variables

In analyzing the database, the primary output variable of interest is the compressive strength of AAC. However, determining the optimal input variables requires a rigorous comparative analysis to identify the most effective configuration. This section outlines the development of four distinct input variable schemes (Options 1~4), each varying in complexity and detail, to improve the prediction accuracy of AAC compressive strength.

Option 1: The first input variable scheme includes 16 variables focusing primarily on the types of raw materials without considering their specific proportions [52,53,54]. These variables encompass OPC, FA, GGBFS, SF, kaolin, and other cementitious materials by mass; coarse and fine aggregate volumes; SS solution; SH solution; additional water; superplasticizer; curing energy; curing humidity; curing age; and specimen shape [55,56,57]. However, this approach may lead to substantial data redundancy, especially when certain raw materials have zero content in many samples [58,59,60]. Additionally, varying material compositions across experiments can introduce noise, further complicating data analysis and reducing model accuracy [61,62,63]. Consequently, this scheme’s effectiveness is limited by its inability to account for material proportions adequately.

Option 2: To address these limitations, the second input variable scheme refines the data processing. This scheme includes 10 input variables and enhances the representation of gelling materials by converting their compositions into oxides and calculating the relative modulus (as defined previously) of the gelling material [64]. The input variables also include the L/S ratio (the ratio of the sum of the SS solution, SH solution, and additional water to the total weight of the gelling material) and the ratio of the SS solution to the SH solution [65,66,67]. Other variables in this scheme are coarse and fine aggregate volumes, superplasticizer, curing energy, curing humidity, curing age, and specimen shape [52,68]. Despite improvements, this scheme still encounters issues with zero values for the superplasticizer in some samples, which may result from experiments where no superplasticizer was used or incomplete data reporting. Additionally, the conversion of alkali activator ions to oxides in RM calculations requires careful consideration [64].

Option 3: The third input variable scheme, consisting of eight variables, further refines the approach by converting individual ions in the alkali activator to their corresponding oxides [69]. These oxides are then incorporated into the RM calculations along with the oxides from gelling materials. To minimize the impact of superplasticizer data, the scheme uses the W/S ratio instead [65]. This scheme retains the variables from Option 2, including coarse and fine aggregate volumes, curing energy, curing humidity, curing age, and specimen shape. By incorporating a more detailed representation of alkali activator ions, this scheme aims to enhance prediction accuracy.

Option 4: For comparative purposes, the fourth input variable scheme treats each oxide as a separate input variable without calculating the RM [7]. This scheme includes a total of 12 input variables, incorporating the same variables as Option 3 but with a focus on individual oxides.

Each scheme represents a progressive refinement in the representation of input variables, aiming to balance detail and practicality. By evaluating these schemes, we seek to identify the optimal configuration for accurately predicting AAC compressive strength. The comparative analysis will provide insights into the trade-offs between data granularity and model performance, ultimately guiding the selection of the most effective input variable set.

### 4.4. Data Pre-Processing and Evaluation Metrics

Before initiating the modeling process, it was crucial to pre-process the database to ensure the accuracy and reliability of the results. The pre-processing steps included the normalization of input variables and splitting the dataset into distinct subsets.

(1) Normalization of input variables: To ensure that each input variable contributes equally to the model, regardless of its original scale, normalization was applied. This step prevents any input variable with a larger range from disproportionately influencing the model’s performance. StandardScaler was employed for this purpose, which transforms each input variable to have a mean of zero and a standard deviation of one, conforming to a standard normal distribution. This process can be described by Equation (15), which standardizes the data by adjusting it to a common scale. This normalization is essential for achieving a fair comparison and accurate weight assignment across variables.

(15) $$x^{*}=\frac{x-\mu }{\sigma }$$

(2) Dataset splitting: The dataset was divided into three subsets, which were training, test, and validation sets, with a sample size ratio of 3:1:1. This division ensures that the model is trained on a substantial portion of the data, while the remaining data are reserved for evaluating the model’s performance and generalizability. The training set is used for model development, the test set for initial performance evaluation, and the validation set for fine-tuning and final validation.

Following the pre-processing phase, the modeling was performed with a focus on parameter optimization. The 10-fold cross-validation technique was utilized for parameter tuning, with scoring based on the negative mean squared error (neg_mean_squared_error) [70,71,72]. In this technique, the dataset is divided into ten equal parts. Nine parts are used for training and the remaining part is used for testing. This process is repeated ten times, each time with a different part used for testing, and the average performance across these iterations provides an estimate of the model’s accuracy.

To assess model performance, the following several metrics were employed:

- Coefficient of determination (R^2^): Measures the proportion of variance in the dependent variable that is predictable from the independent variables. A higher R^2^ indicates better model performance.
- Root mean square error (RMSE): Represents the square root of the average squared differences between predicted and observed values. Lower RMSE values signify better model accuracy.
- Mean absolute error (MAE): Represents the average absolute differences between predicted and observed values. Lower MAE values indicate better model performance.

The formulas used for these evaluations are as follows:

(16) $$R^{2}={\left[\frac{\sum_{i=1}^{n}(e_{i}-\bar{e})(p_{i}-\bar{p})}{\sqrt{\sum_{i=1}^{n}(e_{i}-\bar{e}{)}^{2}\sum_{i=1}^{n}(p_{i}-\bar{p}{)}^{2}}}\right]}^{2},$$

(17) $$\mathrm{RMSE}=\sqrt{\frac{\sum_{i=1}^{n}{\left(e_{i}-p_{i}\right)}^{2}}{n}},$$

(18) $$\mathrm{MAE}=\frac{\sum_{i=1}^{n}\left|e_{i}-p_{i}\right|}{n},$$

where *n* denotes the number of samples involved in the calculation; *e_i_* and *ē* denote the experimental results of the *i*th sample and the average of all experimental results, respectively; and *p_i_* and $\bar{p}$ denote the predicted results of the *i*th sample and the average of all predicted results, respectively. The better performing models have a higher $R^{2}$ and lower RMSE and MAE values.

## 5. Results and Discussion

### 5.1. Unoptimized and Optimized Models

#### 5.1.1. Unoptimized Models

In this section, we address the performance of models that did not incorporate the four optimization algorithms discussed previously. To ensure a fair comparison, we still employed GridSearchCV and RandomSearchCV for hyperparameter tuning and cross-validation. These models, although not optimized by the aforementioned algorithms, serve as essential baselines.

(1) GridSearchCV: This method systematically explores the parameter space by exhaustively evaluating all possible combinations of parameters within predefined ranges. By training the model with each set of parameters, GridSearchCV identifies the combination that yields the highest accuracy on the validation set. This approach ensures thorough exploration but can be computationally intensive.

(2) RandomSearchCV: In contrast, RandomSearchCV samples the parameter space randomly and evaluates a subset of parameter combinations. This technique often requires fewer computations than GridSearchCV and can be effective for large parameter spaces, though it might miss the optimal parameters if they lie in less sampled regions.

Through these tuning processes, we identified local optimal parameters for each model across the four sets of input variables, detailed in Table 2, Table 3, Table 4 and Table 5.

**Figure 7:**
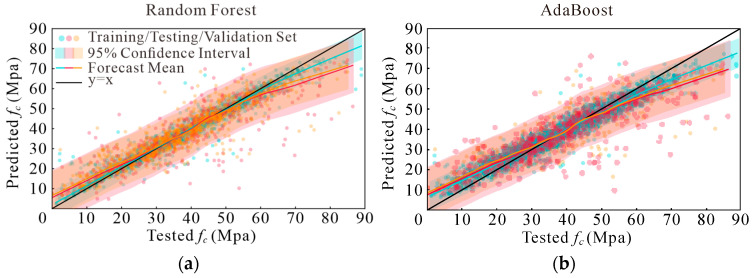

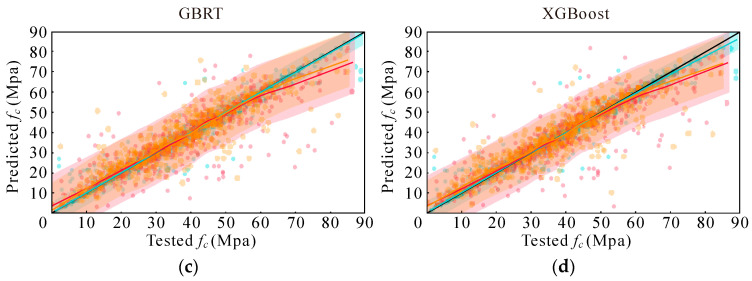
Models’ performance adopting Option 1: (**a**) RF; (**b**) AdaBoost; (**c**) GBRT; and (**d**) XGBoost.

**Figure 8:**
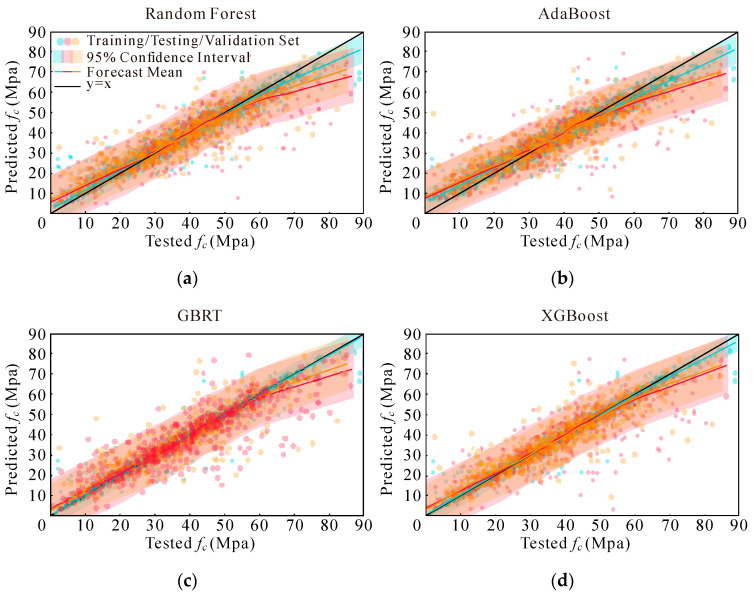
Models’ performance adopting Option 2: (**a**) RF; (**b**) AdaBoost; (**c**) GBRT; and (**d**) XGBoost.

**Figure 9:**
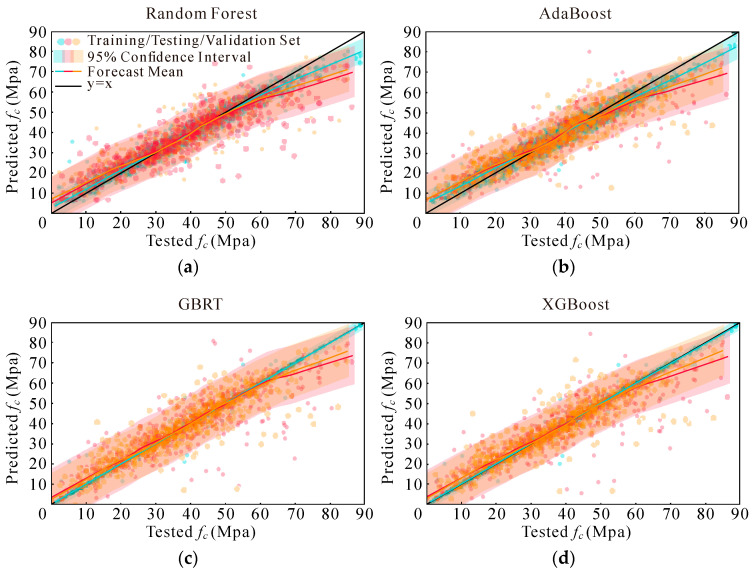
Models’ performance adopting Option 3: (**a**) RF; (**b**) AdaBoost; (**c**) GBRT; and (**d**) XGBoost.

**Figure 10:**
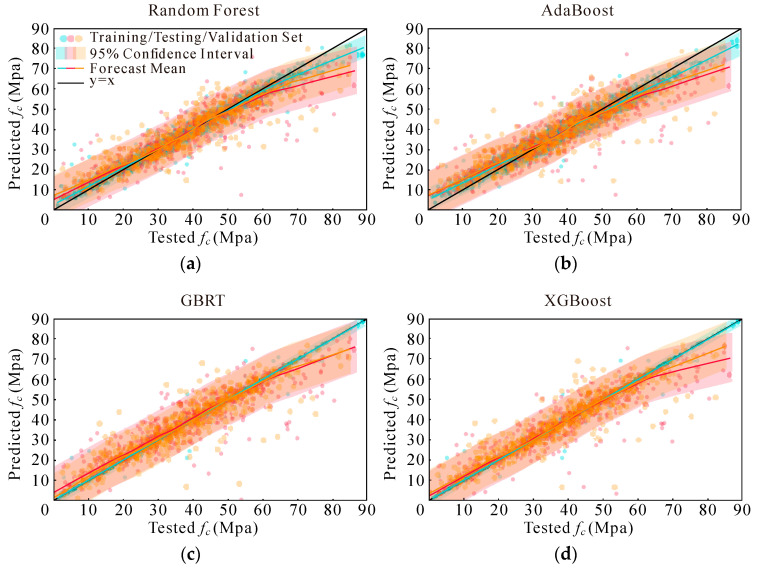
Models’ performance adopting Option 4: (**a**) RF; (**b**) AdaBoost; (**c**) GBRT; and (**d**) XGBoost.

Instead of using basic scatter plots and a reference line (y = x), this study employs a more nuanced visualization approach to assess predicted compressive strengths [69,73]. Figure 7, Figure 8, Figure 9 and Figure 10 present a detailed graphical comparison that includes scatter plots of experimental versus predicted values, the target prediction line (y = x), the best fit line for the data points, and a 95% confidence interval. The training set is denoted in blue, the test set in red, and the validation set in yellow.

The visualizations reveal that the models exhibit high predictive accuracy in the central region of the data distribution, where predictions align closely with observed values. However, discrepancies are evident at both the low and high ends of compressive strength, particularly for values exceeding 60 MPa. Among the models evaluated, XGBoost consistently outperformed RF, while both XGBoost and RF demonstrated better performance compared to AdaBoost and GBRT. Additionally, when comparing the four sets of input variables, the last two sets showed marginal differences. They each outperformed the earlier sets in specific data segments but were uniformly better than the first two sets.

**Figure 11:**
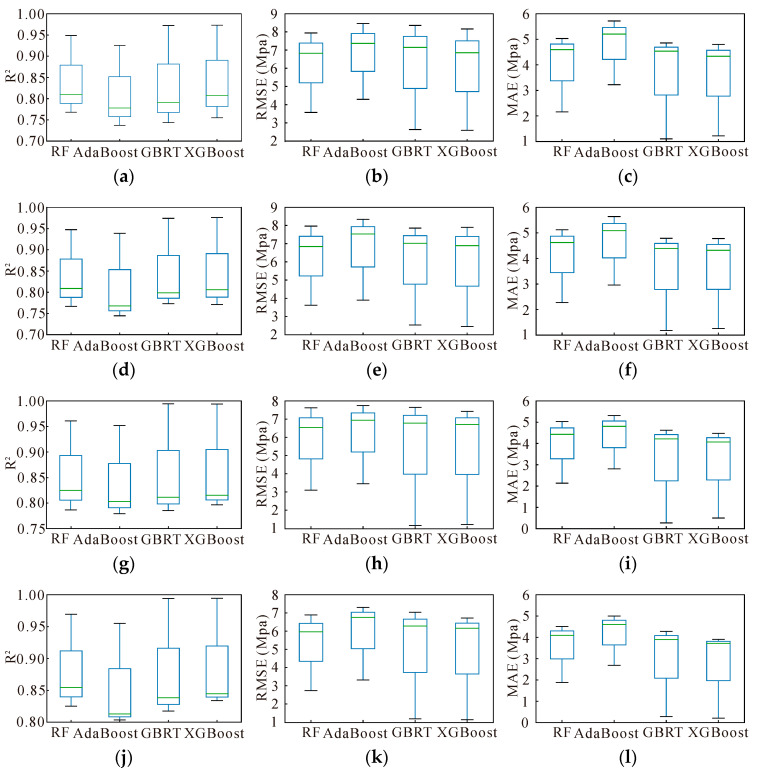
Performance evaluation indicators: (**a**) Option 1—$R^{2}$; (**b**) Option 1—RMSE; (**c**) Option 1—MAE; (**d**) Option 2—$R^{2}$; (**e**) Option 2—RMSE; (**f**) Option 2—MAE; (**g**) Option 3—$R^{2}$; (**h**) Option 3—RMSE; (**i**) Option 3—MAE; (**j**) Option 4-$R^{2}$; (**k**) Option 4—RMSE; and (**l**) Option 4—MAE.

To provide a more comprehensive assessment of model performance, further evaluations were conducted, and the results are summarized in Figure 11. The box plots in Figure 11 indicate that the model built with the fourth set of input variables achieves the best performance across all evaluation metrics, especially in terms of the RMSE and MAE. For detailed insights into evaluation metrics under various conditions, refer to Table 6, Table 7 and Table 8.

The results from Figure 11 and Table 6, Table 7 and Table 8 demonstrate that models utilizing the fourth set of input variables consistently outperform those with other parameter sets. Overall, the XGBoost model, leveraging this optimal set of input variables, shows superior performance compared to RF, AdaBoost, and GBRT. Consequently, the XGBoost model with the fourth set of input variables was selected for further optimization, reflecting its efficacy and robustness in predicting AAC compressive strength.

#### 5.1.2. Optimized Models

As established in the preceding section, the XGBoost model demonstrates the highest initial performance among the evaluated algorithms. To further enhance the predictive accuracy, we applied four advanced optimization algorithms (GWO, WOA, BAS, and BO) to fine-tune the model parameters. This section details the results obtained from each optimization technique and offers a comprehensive comparison.

**Figure 12:**
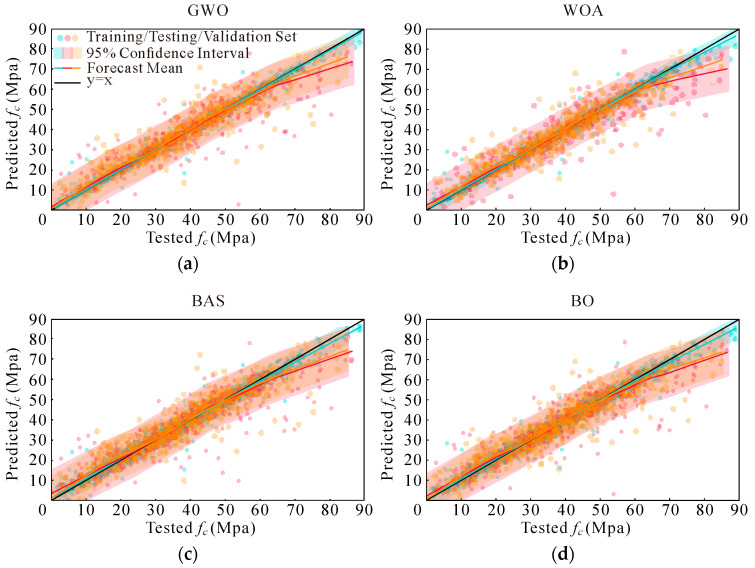
Performance of the best model for each algorithm: (**a**) GWO; (**b**) WOA; (**c**) BAS; and (**d**) BO.

Among the models optimized using these algorithms, the GWO achieved the highest R^2^ value of 0.94 when predicting across the entire dataset. This is followed closely by the WOA, which attained an R^2^ value of 0.93. The models optimized using BAS and BO exhibited R^2^ values of 0.92. These results underscore the effectiveness of the GWO in enhancing model performance. The best models derived from each optimization method are illustrated in Figure 12.

A comparative analysis between Figure 10d and Figure 12 reveals notable insights. Specifically, in the data segment where compressive strength exceeds 60 MPa, the GWO-optimized model shows minimal differences in accuracy on the validation set but demonstrates a significant improvement in accuracy on the test set. This indicates that the GWO enhances model performance in high-strength scenarios more effectively than the other algorithms. Conversely, the WOA-optimized model exhibits stable performance across both validation and test sets, suggesting consistent optimization results. However, models optimized with BAS and BO exhibit a decrease in accuracy on the validation set, while their performance on the test set shows improvement. This variance suggests that while BAS and BO algorithms enhance test set predictions, they may introduce some instability in validation accuracy.

To offer a more precise comparison of each optimization algorithm’s effectiveness, we calculated various evaluation metrics for the optimized models. These metrics are summarized in Table 9. The results indicate that the model optimized using the GWO outperforms all others, exhibiting superior metrics across the board.

Consequently, the GWO-optimized model was selected as the final model for this study. This choice is supported by its superior performance metrics and the overall improvement in predictive accuracy, particularly in scenarios involving high compressive strength. The detailed performance metrics and visualizations provided in Table 9 and Figure 12 substantiate the selection of the GWO as the most effective optimization approach for enhancing XGBoost model accuracy in predicting AAC compressive strength.

### 5.2. Enhancing Model Interpretability

This section delves into the interpretability of the final model, specifically focusing on understanding how different parameters influence model predictions. By utilizing advanced interpretability techniques, including XGBoost’s built-in algorithms and the SHAP library, we aim to shed light on the model’s inner workings, moving beyond the “black box” nature of machine learning models.

#### 5.2.1. Visualizing Model Structure and Feature Importance

**Figure 13:**
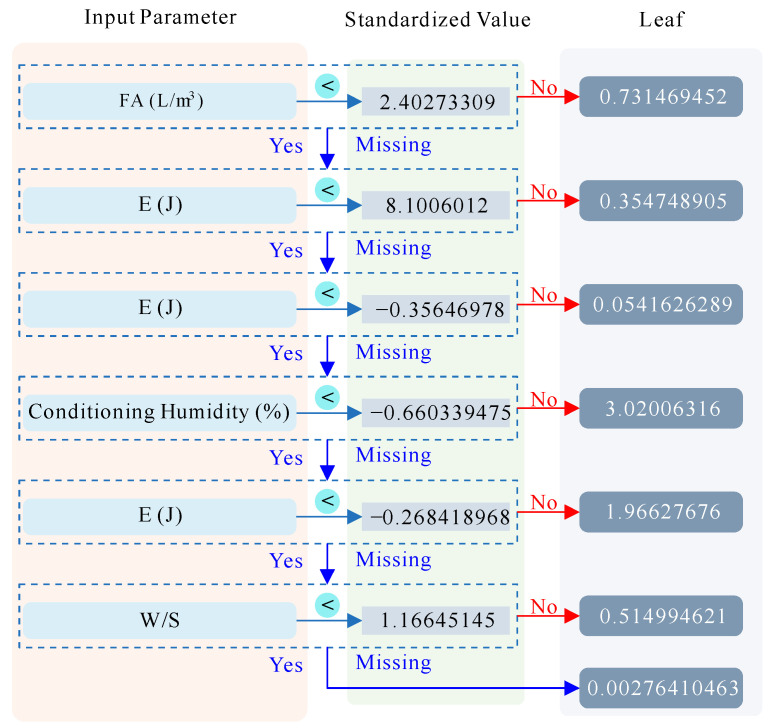
Tree structure made of shallowest leaves.

To gain an initial understanding of the model, we visualized its structure using a decision tree diagram, which provides insights into the model’s decision-making process. The model comprises 675 trees, with the shallowest tree illustrated in Figure 13. This visualization helps us grasp the hierarchical nature of the decision-making process and the distribution of features across different nodes [74].

**Figure 14:**
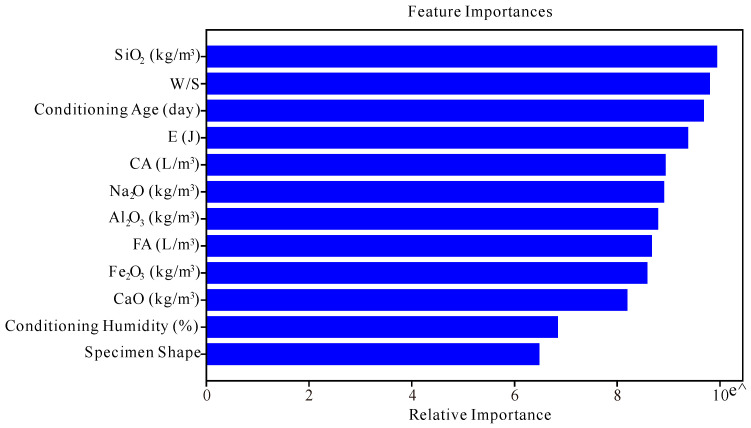
Feature importance.

Feature importance is a critical aspect of model interpretability. It quantifies the contribution of each input variable to the model’s predictive power. In our analysis, feature importance was determined by examining the role of each input variable in constructing the decision trees. Variables with higher importance are those that frequently appear in key decision nodes and contribute significantly to model performance. Figure 14 presents the ranked importance of features, with SiO_2_ emerging as the most influential variable, while the specimen shape is identified as having the least impact.

#### 5.2.2. Interpreting the Model Using SHAP

While feature importance provides a useful overview, it does not fully capture the interactions and nuances of feature contributions. SHAP offers a more granular approach by decomposing the model’s output into contributions from individual features [75,76,77]. SHAP values measure the effect of each feature on the model’s prediction for a given sample, offering both global and local insights into feature importance.

SHAP is an additivity interpretation model. For each sample, the model produces a predictive value, and the Shapley value is the value assigned to each feature in that sample. SHAP simulates the given model *f*(*x*) by constructing a model *g*(*x*) that consists of the base value and the Shapley value of each feature added together. The mathematical equation for model *g*(*x*) is as follows [78]:

(19) $$g\left(x\right)=g_{0}+\sum_{i=1}^{n}g_{i}z_{i},$$

where *n* is the number of features; $z_{i}\in \left\{0,1\right\}$ indicates whether feature *i* can be observed, and features that cannot be observed will not affect the interpretation; $g_{0}$ is a constant in the explanation model, and its value equals the mean prediction of all training samples; and $g_{i}\in \mathbb{R}$ is the Shapley value, which can be calculated from Equation (20).

(20) $$g_{i}\left(f,x\right)=\sum_{z\subseteq {N\backslash x}_{i}}\frac{\left|z\right|!\left(n-\left|z\right|-1\right)!}{n!}\left[f\left(N\right)-f\left(z\right)\right],$$

where *z* denotes the subset of features (excluding feature *i*), and *N* represents the total set of features.

**Figure 15:**
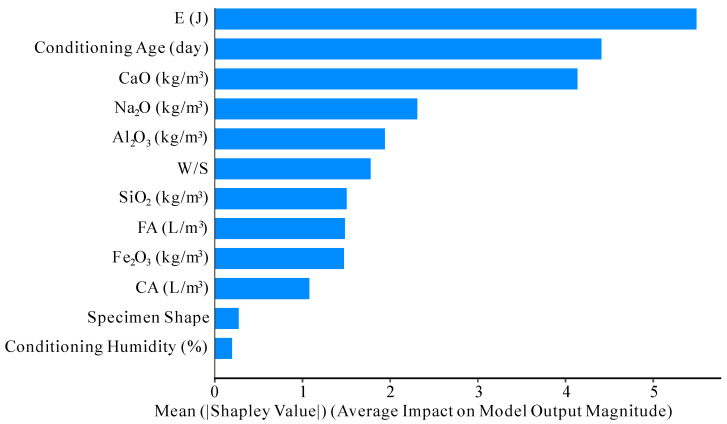
Shapley value.

When the model is analyzed using SHAP, the importance of each feature in the model is first output. The importance diagram is shown in Figure 15.

Compared to the feature importance determined using the built-in XGBoost algorithm in Figure 14, the Shapley values shown in Figure 15 are more suitable for a comprehensive analysis of the model. The feature importance calculated by the XGBoost built-in algorithm primarily relies on the usage of features within the tree structure. This assessment includes aspects such as the frequency of features appearing in splits, gain (the increase in purity brought about by each feature split), and coverage (the number of samples covered by each feature split). However, because this method is based on the structure of the model, it cannot accurately capture feature interactions and nonlinear effects. Therefore, we use SHAP to further calculate the marginal contribution of each feature to the model output, providing a more accurate measure of feature importance. SHAP takes into account all possible combinations and interactions of features, offering both global and local interpretations of feature impact on predictions [79].

As is evident from both Figure 14 and Figure 15, the energy consumed during curing and the curing age are among the most influential factors affecting the compressive strength of AAC. In contrast, the shape of the specimen and the curing humidity are ranked among the least impactful factors on the compressive strength in the model.

**Figure 16:**
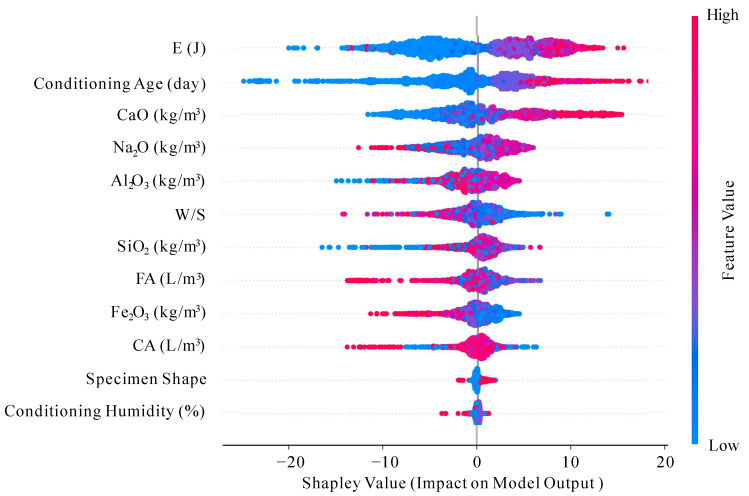
Global Shapley value distribution.

Figure 15 visualizes the importance of each feature within the model; however, it does not explicitly illustrate the relationship between each feature and the model’s predictions. To gain a more comprehensive understanding, we need to further analyze the global impact of the input variables across the entire dataset [80], as shown in Figure 16.

Since this model is not a universally applicable model, the results obtained through SHAP analysis may not always correspond to real-world scenarios. Therefore, we focused only on analyzing the data that exhibited clearer patterns.

In Figure 16, it can be seen intuitively how various features affect the model. For instance, to some extent, higher curing energy values, curing age, and CaO content lead to higher model predictions, while a higher fine aggregate volume and Fe_2_O_3_ content result in lower model predictions.

**Figure 17:**
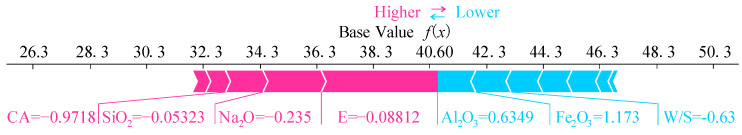
The contribution of features.

SHAP can also be used to analyze the contribution of features for each sample individually. For example, Figure 17 shows the contribution of features for one sample in the dataset.

The mechanism by which each feature operates in predicting this sample can be clearly seen from the individual feature contribution plots.

**Figure 18:**
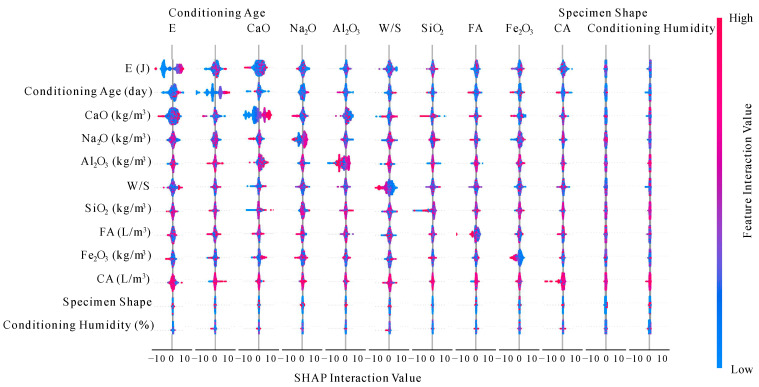
The interactions between the input variables.

In addition to representing the effect of individual features on the overall model or individual predicted values, SHAP can also analyze the interaction of multiple variables. Figure 18 shows the interactions between the input variables.

In Figure 18, the effect of each feature’s SHAP value on its own interaction attribution value is represented on the diagonal, and the effect of each feature’s SHAP value on the interaction attribution values of other features is represented off the diagonal. From Figure 18, it can be seen that both specimen shape and curing humidity have a small degree of interaction with each feature and do not affect the model and each feature to a significant degree.

**Figure 19:**
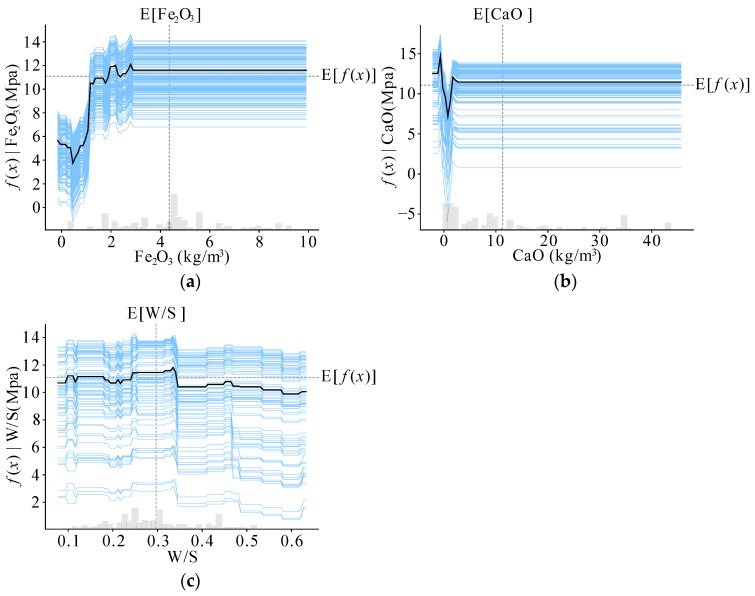
Individual conditional expectation plots: (**a**) Fe_2_O_3_; (**b**) CaO; and (**c**) W/S.

To better observe the global impact of each input variable on the output parameter, ICE plots were created, as shown in Figure 19. Only some of the input variables that are globally more variable in their effect on the model output parameters have been selected to be represented in Figure 19.

From Figure 19, it is evident that Fe_2_O_3_ and CaO show considerable variation in their impact on model predictions at lower concentrations, with their influence stabilizing at higher levels. This behavior may be due to incomplete reactions of raw materials at higher concentrations or limitations in the dataset.

In summary, our analysis using SHAP and other interpretability techniques provides a clearer understanding of how different parameters influence the model’s predictions. By elucidating the contributions and interactions of features, we enhance the transparency and reliability of the model, facilitating more informed decisions in the context of geopolymers and machine learning integration.

## 6. Conclusions and Further Research

To enhance the prediction accuracy of compressive strength in the AAC manufacturing process, this study developed a robust predictive model based on an extensive dataset derived from 111 studies. The constructed model aims to accurately forecast AAC compressive strength, offering valuable insights for AAC manufacturing. Unlike conventional approaches, this study prioritized a careful selection of input variables, which guided the development and optimization of the model. The process involved a systematic evaluation of four distinct input variable sets, four ML models, and four optimization algorithms, ultimately leading to the identification of the most effective model. Subsequent analysis focused on understanding the impact of each input variable on the model’s predictions.

Based on the in-depth analysis conducted throughout this study, the following conclusions have been drawn:

(1) The accuracy and effectiveness of predictive models heavily depend on the selection of input variables. It is essential to prioritize variables related to the chemical composition of the reactants (e.g., the relative modulus), which significantly influence the reaction process. Variables with minimal impact should be excluded to prevent them from diluting the model’s performance. The accurate and comprehensive representation of influential factors is crucial for enhancing model precision;
(2) Among the four machine learning models evaluated—RF, AdaBoost, GBRT, and XGBoost—the XGBoost model demonstrated superior performance across all scenarios. XGBoost consistently outperformed the other models, proving to be the most reliable for predicting AAC compressive strength. In comparison, RF exhibited slightly lower performance, while AdaBoost and GBRT yielded the least favorable results;
(3) Of the four optimization algorithms applied—the GWO, WOA, BAS, and BO—the GWO achieved the highest performance, with an R^2^ value of 0.94 for the entire dataset. The WOA followed closely, with an R^2^ of 0.93, while BAS and BO recorded maximum R^2^ values of 0.92. These results underscore the superior optimization capabilities of these algorithms in enhancing model accuracy;
(4) The integration of XGBoost’s built-in algorithms and SHAP library proved instrumental in analyzing feature importance and model interpretability. SHAP provides a comprehensive understanding of feature contributions by quantifying the marginal impact of each feature on the model’s predictions. This analysis revealed that curing energy and curing age are significant factors affecting compressive strength, while specimen shape and curing humidity have relatively minor impacts. Additionally, the analysis highlighted that a higher curing energy generally leads to increased model predictions, whereas a higher volume of fine aggregate correlates with lower predictions. Notably, Fe_2_O_3_ and CaO demonstrated substantial variability in impact at lower concentrations, with their influence stabilizing at higher levels. This variability could be attributed to incomplete reactions or insufficient sample data, suggesting the need for further refinement of the model.

Machine learning, with its capacity to analyze extensive datasets, holds the potential to transform materials science by uncovering complex patterns and relationships that traditional analytical methods might miss. The predictive model for AAC compressive strength developed here represents a certain advancement in predictive analytics, offering a robust tool for estimating compressive strength based on various input parameters. By harnessing the stochastic nature of optimization algorithms, this model generates multiple predictive outcomes and corresponding R^2^ values, providing a thorough reference for AAC compressive strength prediction. The code for this model is now available at https://github.com/xbmhmy/AACcode (accessed on 3 September 2024).

It is crucial to acknowledge that the compressive strength data within this study’s database predominantly fall within the 30–60 MPa range. Consequently, the model exhibits high predictive accuracy within this interval, whereas predictions for values outside this range demonstrate decreased accuracy. Due to the inherent complexity of AAC compositions, our model currently cannot flawlessly predict the strength of all AAC formulations. To address this limitation and enhance the model’s robustness, expanding the AAC database and incorporating advanced machine learning techniques, evolving optimizers like Optuna, and domain knowledge-infused methods, are essential to improving both accuracy and reliability.

Nonetheless, the integration of machine learning into materials science offers the potential to advance our understanding of material properties and performance. By leveraging sophisticated data-driven algorithms, machine learning can uncover hidden correlations and predictive factors that are not immediately apparent. We anticipate that a broader incorporation of cement chemistry knowledge into machine learning frameworks will facilitate this integration. This interdisciplinary approach could lead to a deeper understanding of how various chemical and physical parameters affect AAC compressive strength, thereby improving the model’s accuracy and applicability.

Furthermore, combining machine learning with experimental research can refine the optimization of AAC mix proportions. By simultaneously enhancing the model and optimizing mix designs, researchers can achieve more accurate control over raw material selection and environmental conditions. This dual approach not only improves the model’s performance but also stimulates innovations in AAC production processes. Future studies should also investigate the internal mechanisms and interpretability of related machine learning models to develop more actionable insights, ultimately advancing materials science and engineering practices.

Through these efforts, machine learning can infuse materials science with renewed dynamism and research vitality, offering novel perspectives and solutions that push the boundaries of current knowledge and applications.

## Figures and Tables

**Table 1:** Summary of parameters.

Parameters	Detailed Meaning	Unit	Range
Cement	The mass of cement per unit volume of AAC	kg/m^3^	[0, 270)
FA	The mass of FA per unit volume of AAC	kg/m^3^	[0, 640]
GGBFS	The mass of GGBFS per unit volume of AAC	kg/m^3^	[0, 560]
SF	The mass of silica fume (SF) per unit volume of AAC	kg/m^3^	[0, 270]
Kaolin	The mass of kaolin per unit volume of AAC	kg/m^3^	[0, 400)
Other SCM	The mass of other supplementary cementitious materials (SCM) per unit volume of AAC	kg/m^3^	[0, 450]
SiO_2_	The mass of SiO_2_ within the cementitious materials per unit volume of AAC	kg/m^3^	(30, 180)
Al_2_O_3_	The mass of Al_2_O_3_ within the cementitious materials per unit volume of AAC	kg/m^3^	(4, 40)
Fe_2_O_3_	The mass of Fe_2_O_3_ within the cementitious materials per unit volume of AAC	kg/m^3^	(0, 19)
CaO	The mass of CaO within the cementitious materials per unit volume of AAC	kg/m^3^	(0, 50)
Na_2_O	The mass of Na_2_O within the cementitious materials per unit volume of AAC	kg/m^3^	(3, 55)
RM	Relative modulus	–	(0, 1.1)
CA	The volume of coarse aggregate per unit volume of AAC	L/m^3^	[0, 600)
FA	The volume of fine aggregate per unit volume of AAC	L/m^3^	[0, 430)
Na_2_SiO_3_ (l)	The mass of Na_2_SiO_3_ (liquid) in the superplasticizer per unit volume of AAC	kg/m^3^	(20, 370)
NaOH (l)	The mass of NaOH (liquid) in the superplasticizer per unit volume of AAC	kg/m^3^	(5, 240)
SS/SH	The ratio of the mass of SS to the mass of SH	–	(0, 15)
Additional water	The mass of additional water per unit volume of AAC	kg/m^3^	[0, 220)
Superplasticizer	The mass of superplasticizer per unit volume of AAC	kg/m^3^	[0, 120)
L/S	The ratio of the sum of the content of the SS solution, SH solution, and additional water to the total weight of the gelling material	–	(0, 1.6)
W/S	The ratio of the sum of the SS solution, SH solution, additional water, and superplasticizer content to the total weight of the cementitious material	–	(0, 1)
E	The total energy consumed per unit weight of AAC during curing	J	[−4.2 × 10^7^, 2.2 × 10^9^)
Curing humidity	Curing humidity of the specimen	%	[20, 100]
Curing age	Curing age of the specimen	day	[1, 365]
Specimen shape	Specimen shape: prism or cylinder	–	1/2

**Table 2:** RF parameters.

	Option 1	Option 2	Option 3	Option 4
n_estimators	435	876	818	821
max_depth	31	33	24	31
max_leaf_nodes	719	690	550	550
min_samples_leaf	2	2	1	1
min_samples_split	2	2	6	5
bootstrap	False	False	False	False
random_state	0	0	0	0
max_features	6	4	3	4

**Table 3:** AdaBoost parameters.

	Option 1	Option 2	Option 3	Option 4
max_depth	24	16	20	15
min_samples_split	2	2	2	2
min_samples_leaf	3	2	3	3
max_leaf_nodes	250	255	260	265
criterion	mse	mse	mse	mse
n_estimators	310	295	285	300
learning_rate	0.1	0.1	0.1	0.11
random_state	0	0	0	0

**Table 4:** GBRT parameters.

	Option 1	Option 2	Option 3	Option 4
n_estimators	136	132	355	356
learning_rate	0.21	0.22	0.16	0.15
max_depth	53	48	57	51
min_samples_leaf	9	10	7	7
min_samples_split	2	2	2	2
random_state	0	0	0	0
loss	ls	ls	ls	ls

**Table 5:** XGBoost parameters.

	Option 1	Option 2	Option 3	Option 4
max_depth	28	26	27	27
learning_rate	0.01	0.01	0.03	0.02
n_estimators	736	735	735	736
colsample_bytree	1	1	1	1
subsample	0.76	0.73	0.77	0.77
gamma	2	3.1	1	0
random_state	0	0	0	0

**Table 6:** Performance evaluation indicators—training.

	Option 1	Option 2	Option 3	Option 4
RF-$R^{2}$	0.95	0.95	0.96	0.97
AdaBoost-$R^{2}$	0.93	0.94	0.95	0.96
GBRT-$R^{2}$	0.97	0.97	0.99	0.99
XGBoost-$R^{2}$	0.97	0.98	0.99	0.99
RF-RMSE (MPa)	3.57	3.61	3.11	2.75
AdaBoost-RMSE (MPa)	4.30	3.90	3.46	3.34
GBRT-RMSE (MPa)	2.63	2.53	1.18	1.20
XGBoost-RMSE (MPa)	2.59	2.44	1.23	1.15
RF-MAE (MPa)	2.16	2.27	2.14	1.89
AdaBoost-MAE (MPa)	3.22	2.96	2.81	2.68
GBRT-MAE (MPa)	1.10	1.18	0.27	0.28
XGBoost-MAE (MPa)	1.22	1.26	0.51	0.20

**Table 7:** Performance evaluation indicators—testing.

	Option 1	Option 2	Option 3	Option 4
RF-$R^{2}$	0.77	0.77	0.79	0.83
AdaBoost-$R^{2}$	0.74	0.74	0.78	0.80
GBRT-$R^{2}$	0.74	0.77	0.79	0.82
XGBoost-$R^{2}$	0.76	0.77	0.80	0.83
RF-RMSE (MPa)	7.94	7.97	7.62	6.90
AdaBoost-RMSE (MPa)	8.46	8.34	7.75	7.31
GBRT-RMSE (MPa)	8.35	7.86	7.64	7.05
XGBoost-RMSE (MPa)	8.16	7.90	7.43	6.72
RF-MAE (MPa)	5.03	5.12	5.03	4.51
AdaBoost-MAE (MPa)	5.71	5.64	5.31	5.00
GBRT-MAE (MPa)	4.85	4.79	4.62	4.28
XGBoost-MAE (MPa)	4.80	4.78	4.48	3.90

**Table 8:** Performance evaluation indicators—validation.

	Option 1	Option 2	Option 3	Option 4
RF-$R^{2}$	0.81	0.81	0.83	0.85
AdaBoost-$R^{2}$	0.78	0.77	0.80	0.81
GBRT-$R^{2}$	0.79	0.80	0.81	0.84
XGBoost-$R^{2}$	0.81	0.81	0.82	0.84
RF-RMSE (MPa)	6.83	6.84	6.54	5.97
AdaBoost-RMSE (MPa)	7.37	7.54	6.94	6.76
GBRT-RMSE (MPa)	7.14	7.02	6.79	6.29
XGBoost-RMSE (MPa)	6.86	6.89	6.72	6.16
RF-MAE (MPa)	4.59	4.62	4.43	4.09
AdaBoost-MAE (MPa)	5.20	5.09	4.80	4.61
GBRT-MAE (MPa)	4.53	4.39	4.21	3.89
XGBoost-MAE (MPa)	4.34	4.32	4.07	3.72

**Table 9:** Performance evaluation indicators.

	Train	Test	Validation	All Data
GWO-$R^{2}$	0.99	0.85	0.87	0.94
WOA-$R^{2}$	0.98	0.86	0.87	0.93
BAS-$R^{2}$	0.97	0.84	0.87	0.92
BO-$R^{2}$	0.97	0.84	0.86	0.92
GWO-RMSE (MPa)	1.67	6.32	5.60	3.99
WOA-RMSE (MPa)	2.01	6.26	5.69	4.09
BAS-RMSE (MPa)	2.51	6.67	5.74	4.39
BO-RMSE (MPa)	2.54	6.53	5.87	4.39
GWO-MAE (MPa)	0.84	3.69	3.51	1.94
WOA-MAE (MPa)	1.03	3.70	3.61	2.08
BAS-MAE (MPa)	1.75	4.21	3.83	2.66
BO-MAE (MPa)	1.70	4.08	3.94	2.62

## Data Availability

The original contributions presented in the study are included in the article, further inquiries can be directed to the corresponding author.
